# Supplementary material for: A novel humanized mouse model to study the function of human cutaneous memory T cells in vivo in human skin
Source: Sci Rep. 2020 Jul 7;10:11164. doi: 10.1038/s41598-020-67430-7 (PMC7341892; doi:10.1038/s41598-020-67430-7)
Supplement: Supplementary file 1 — Supplementary file1 [file 41598_2020_67430_MOESM1_ESM.pdf]

# Supplementary data

## A novel humanized mouse model to study the function of human cutaneous memory T cells *in vivo* in human skin

**Short title:** ***Cutaneous T cells in a skin-humanized mouse model***

Maria M. Klicznik[1], Ariane Benedetti[1], Laura M. Gail[1], Suraj R. Varkhande[1], Raimund Holly[1], Martin Laimer[2], Angelika Stoecklinger[1], Andreas Sir[3], Roland Reitsamer[3], Theresa Neuper[1], Jutta Horejs-Hoeck[1], Michael D. Rosenblum[4], Daniel J. Campbell[5], Eva M. Murauer[6], Iris K. Gratz[1,5,6]

[1] Department of Biosciences, University of Salzburg, Salzburg, Austria
[2] Department of Dermatology, University Hospital of the Paracelsus Medical University Salzburg, Austria
[3]Breast Center, University Hospital of the Paracelsus Medical University Salzburg, Austria
[4]Department of Dermatology, University of California, San Francisco CA 94143, USA.
[5]Benaroya Research Institute, 1201 9th AVE, Seattle, WA 98101 USA
[6]Division of Molecular Dermatology and EB House Austria, Department of Dermatology, Paracelsus Medical University Salzburg, Austria

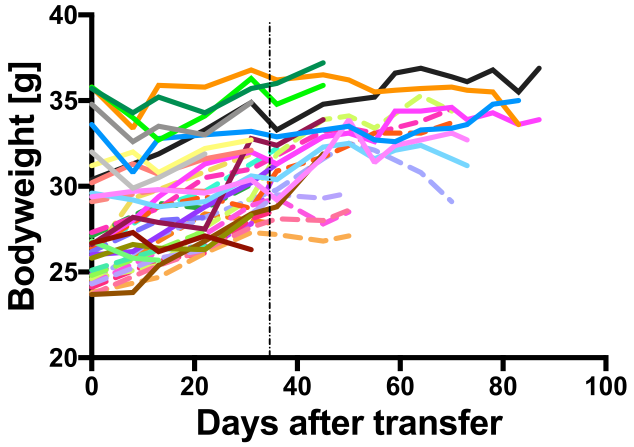


**Supplementary Figure 1: Body weight curves of experimental mice after adoptive transfer human PBMC.** NSG mice were adoptively transferred with human PBMC and weight was measured at indicated time points. Graph shows 2 representative experiments of 2 different PBMC donors (full lines and dotted lines) after adoptive transfer of 2.5x$10^6$ PBMC; n=36. Vertical black dotted line represents the limit of 35 days for all further experiments using huPBMC-ES-NSG mice.

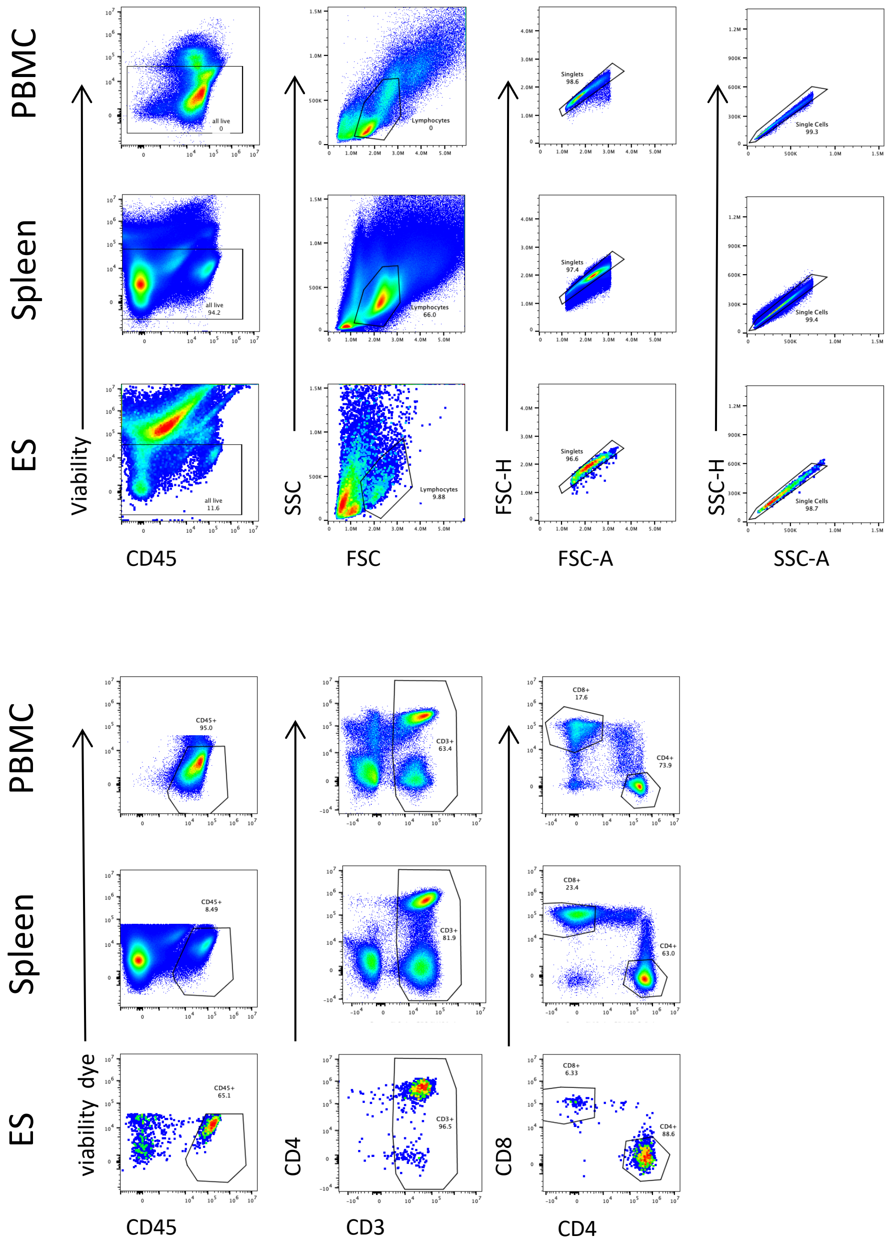


**Supplementary Figure 2: Gating strategy for flow cytometric analysis of human PBMC, spleen and ES single-cell suspensions**

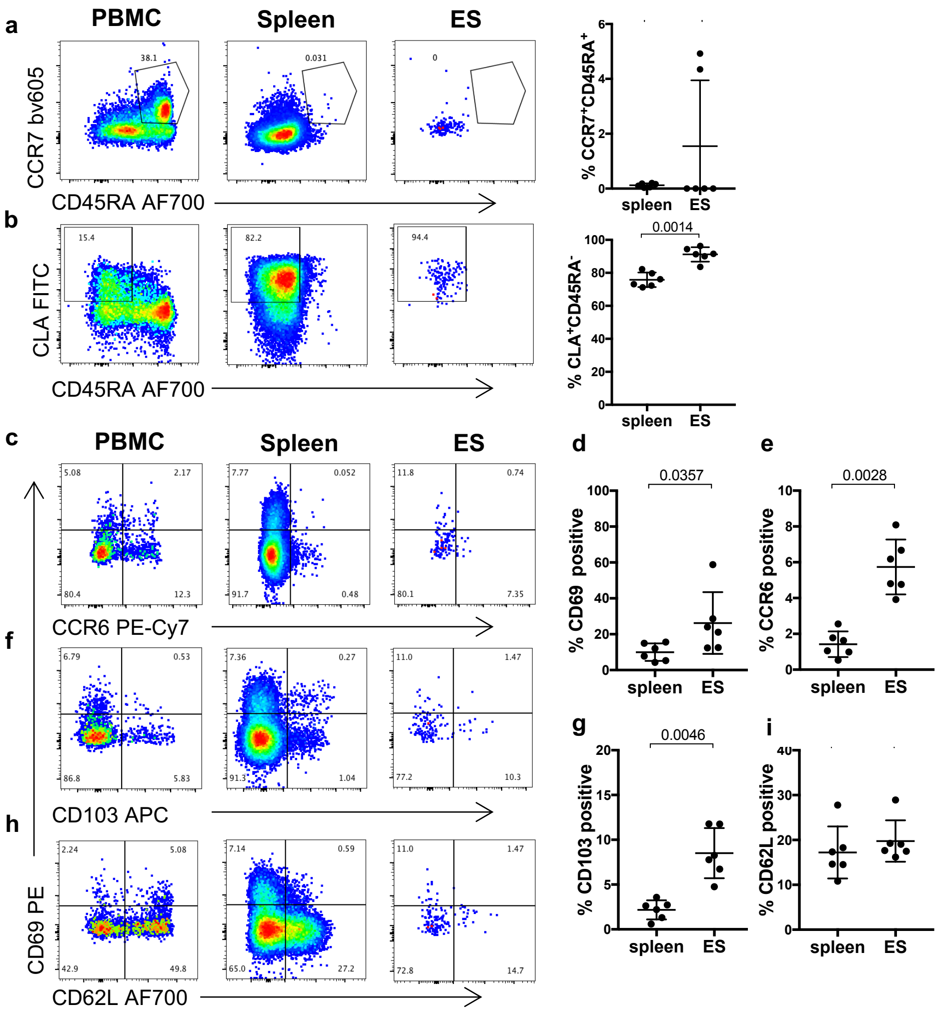


**Supplementary Figure 3: Skin and spleen infiltrating CD8$^{+}$ T cells show skin-homing memory phenotype and upregulate markers of tissue residency and skin-tropism in human ES.** Representative flow cytometry analysis of (**a**) CCR7 and CD45RA expression, and (**b**) CLA and CD45RA expression by gated CD4$^{+}$CD3$^{+}$CD45$^{+}$ live leukocytes from blood of healthy donors, spleen and ES of huPBMC-ES-NSG mice and graphical summary of the proportions of indicated cells by gated CD4$^{+}$CD3$^{+}$CD45$^{+}$ live leukocytes. n=5-6/experiment; cumulative data of 2 independent experiments. (**c, f, h**) Representative flow cytometry analysis for expression of (**c**) CCR6 and CD69; (**f**) CD103 and (**h**) CD62L in indicated tissues by gated CLA$^{+}$CD45RA$^{-}$CD4$^{+}$CD3$^{+}$ live cells from blood of healthy donors, spleen and ES of huPBMC-ES-NSG mice. (**d, e, g, i**) Graphical summary of the expression of the indicated markers by CLA$^{+}$CD45RA$^{-}$CD4$^{+}$CD3$^{+}$living cells isolated from spleen and ES. Each dot represents an individual animal; Significance determined by paired student's t test; mean $^{+}$/- SD

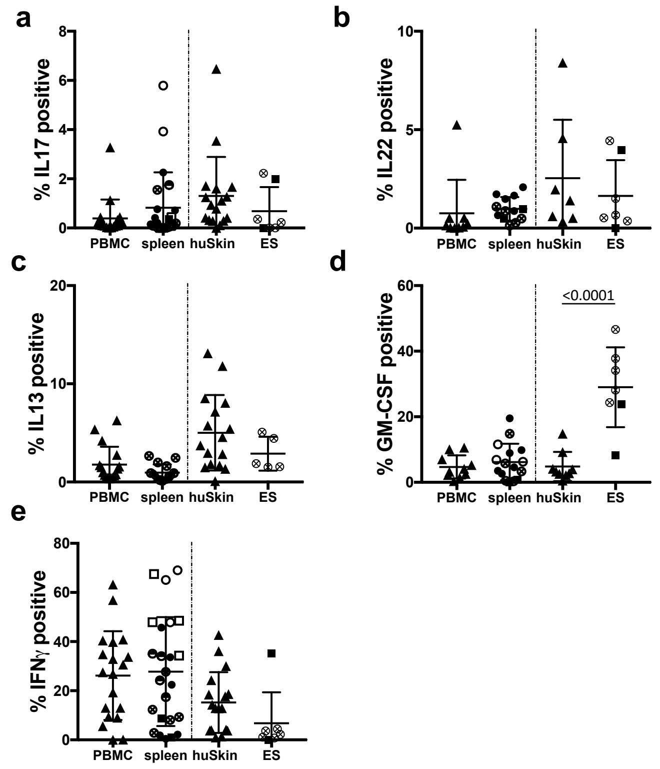


**Supplementary Figure 4: Engrafted splenic and cutaneous human $CD8^+$ T cells reflect diverse phenotypes of T cells in human tissues.** Single cell suspensions of blood and skin of healthy donors, and spleen and ES of huPBMC-ES-NSG mice were prepared 18-35 days after PBMC transfer, stimulated *ex vivo* with PMA/ionomycin and intracellular cytokine production was analyzed by flow cytometry. (**a-e**) Graphical summary of flow cytometry analysis of IL17, IL22, IL13, GM-CSF and IFNγ producing $CD8^+CD3^+CD45^+$ gated live leukocytes from blood and skin of healthy donors and spleen and ES of huPBMC-ES-NSG mice as indicated upon *ex vivo* stimulation with PMA/Ionomycin and intracellular staining. n=3-6/ experiment; combined data of 1-4 independent experiments, as indicated by the different fillings of the symbols.

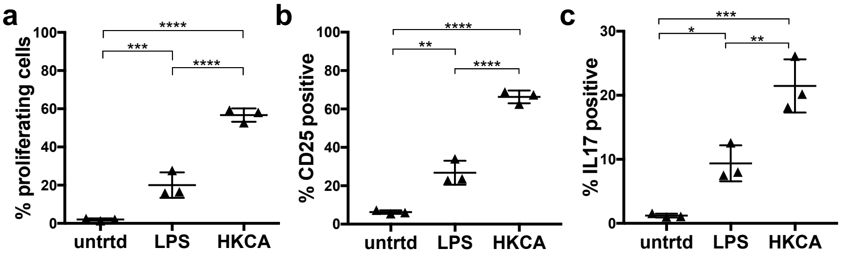


**Supplementary Figure 5: Heat killed *C.alibicans* presented by moDCs leads to proliferation and activation of CD4$^+$ T cells.** PBMC were cultured *in vitro i*n the presence of monocyte-derived DCs (moDC) for 7 days and the proliferation, CD25 expression and cytokine production of CD4$^+$ T cells gated by CD3$^+$CD45$^+$live leukocytes was assessed by flow cytometry. (**a**) Graphical summary of proliferating CD4$^+$ T cells and expression of the indicated markers by of human PBMC with autologous non-activated moDC (untrd), LPS loaded moDC (LPS) or HKCA loaded moDC (HKCA). PBMC were stained with the cell proliferation dye eFluor 450 to identify proliferating cells. n=3/group. P values indicated as asterisk: * ≤ 0.05; ** ≤ 0.01, *** ≤ 0.001, **** ≤ 0.0001

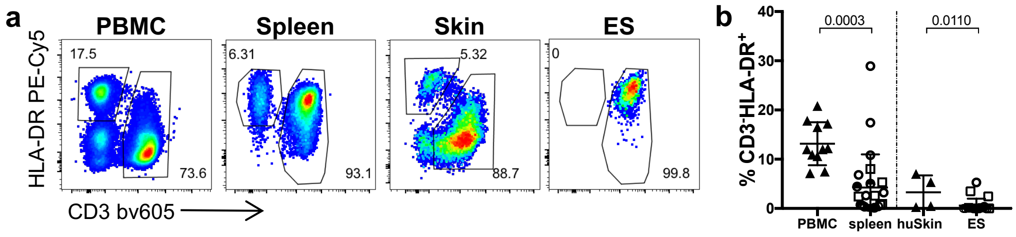


**Supplementary Figure 6: CD3⁻HLA-DR⁺ antigen presenting cells do not engraft well within huPBMC-ES-NSG. a**) Representative flow cytometry analysis of $CD3^-HLA\text{-}DR^+$ cells in human PBMC, skin and spleen and ES of huPBMC-ES-NSG and (**b**) graphical summary of $CD3^-HLA\text{-}DR^+$ cells gated on live $CD45^+$ leukocytes. n=4-6/group, combined data of 1-5 independent experiments, as indicated by the fillings of the symbols.

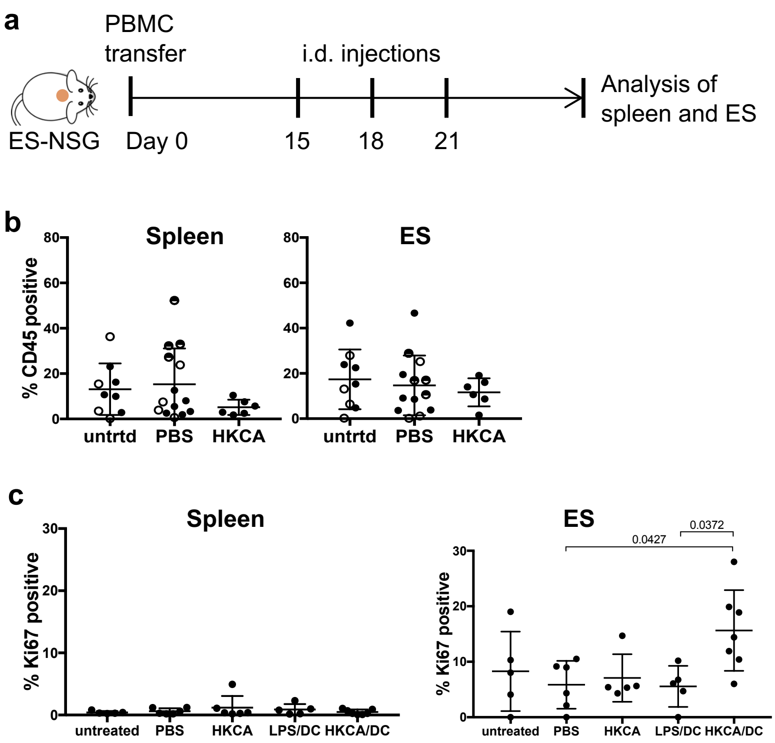


**Supplementary Figure 7: Free heat killed *C.alibicans* fails to elicit proliferation and activation of $CD4^+$ T cells.** (**a**) Experimental procedure of the injection of $1x10^6$/ml free HKCA cells into the ES. Single cell suspensions of indicated organs were analyzed by flow cytometry. (**b**) Graphical summary of human $CD45^+$ cell proportions of live leukocytes in spleen and ES of mice that were left untreated (untrtd), injected with PBS or free HKCA. n=3-5/group, combined data of 1-3 independent experiments Statistical significance determined by ANOVA and Tuckey's test for multiple comparison; mean +/-SD. (**c**) The ES of huPBMC-ES-NSG mice were injected with PBS, free HKCA, LPS-activated moDC or HKCA-loaded moDC and harvested 7 days after the last injection similar to (a). Graphical summary of the fraction of $Ki67^+$ $CD4^+$ cells within the spleen and ES after i.d. injections of the ES as indicated, gated on viable $CD45^+CD3^+$ cells.

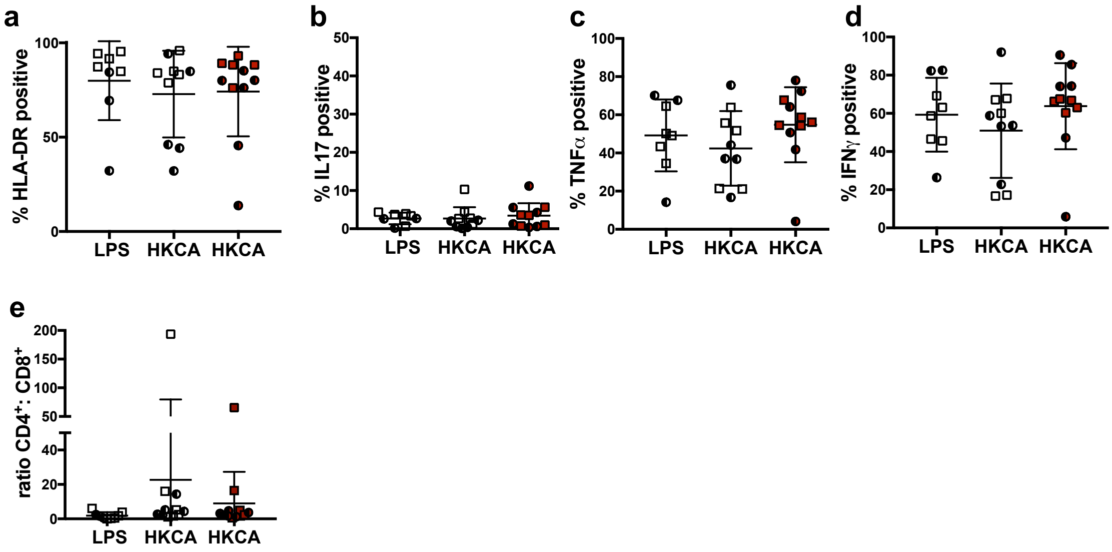


**Supplementary Figure 8: Splenic T cell are not activated by an allogeneic ES or by HKCA presented in the ES.**
NSG mice bearing fully healed ES of one of two different skin donors (A and B) were adoptively transferred with either skin donor-matched PBMC or skin donor-mismatched PBMC. Intradermal injections of donor A derived LPS/moDC or HKCA/moDC were performed as depicted in Fig. 6 (i.e. leukocytes were matched). Single cell suspensions of spleens were analyzed by flow cytometry after *ex vivo* stimulation with PMA/Ionomycin and intracellular staining. (**a**) Graphical summary of the expression of HLA-DR by $CD4^+$ T cells of $CD3^+CD45^+$ live leukocytes isolated from the spleen of huPBMC-ES-NSG mice after intradermal injection of LPS/moDC (LPS) or HKCA/moDC (HKCA) into ES matched (black or empty symbols) or mismatched (red symbols) to the leukocytes. (**a-d**) Splenocytes were stimulated with PMA/ionomycin *ex vivo* and intracellular cytokine production of the indicated cytokines analyzed by flow cytometry. (**e**) Ratio of $CD4^+$ to $CD8^+$ cells in spleens of huPBMC-ES-NSG mice, gated on $CD3^+CD45^+$ live leukocytes. Each symbol represents a single animal, data compiled from 2-5 independent experiments as indicated by the fillings of the symbols.

## Table S1: Detailed list of antibodies and reagents

**Tissue preparation**

| Reagent | Company | Catalog number |
|---|---|---|
| Collagenase Type 4 | Worthington | LS004186 |
| DNAse | Sigma-Aldrich | DN25 |
| RPMI 1640 | Gibco | 31870074 |
| human serum | Sigma-Aldrich | H5667/H4522 |
| Penicillin/streptavidin | Sigma-Aldrich | P0781 |
| L-Glutamine | Gibco | A2916801 |
| NEAA | Gibco | 11140035 |
| Sodium-Pyruvat | Sigma-Aldrich | S8636 |
| β-Mercaptoethanol | Gibco | 31350-010 |
| PBS | Gibco | 14190169 |
| Ficoll Paque Plus | GE-Healthcare | GE17-1440-02 |
| Protease Inhibitor Cocktail | Sigma-Aldrich | P8340 |

**Cellular activation**

| Reagent | Company | Catalog number |
|---|---|---|
| Brefeldin A | Sigma-Aldrich | B6542 |
| Cytofix/Cytoperm kit | BD | RUO 554714 |
| Foxp3 / Transcription Factor Staining Buffer Set | Invitrogen | 00-5523-00 |
| Ionomycin | Sigma-Aldrich | I06434 |
| PMA | Sigma-Aldrich | P8139 |
| Recombinant human IL2 | Immunotools | 11340023 |
| Recombinant human IL4 | Immunotools | 11340047 |
| Recombinant human GM-CSF | Immunotools | 11343127 |
| Purified NA/LE Mouse anti-human CD28 (CD28.2) | BD | 555725 |
| Functional Grade, CD3 monoclonal Antibody (OKT3) | eBioscience | 16-0037-85 |
| Cytokine/Chemokine/Growth Factor 45-Plex Human ProcartaPlex™ | Invitrogen | EPX-450-12171-901 |

**Skin cell culture and transplantation**

| Reagent | Company | Catalog number |
|---|---|---|
| Epilife | Gibco | MEPICF500 |
| DMEM | Gibco | 11960-044 |
| MEM | Gibco | 11380037 |
| TrypLE express | Gibco | 12604021 |
| Primocin | invitrogen | ant-pm-1 |
| X-VIVO 15 | Lonza | 881028 |

**Tissue preparation from mice**

| Reagent | Company | Catalog number |
|---|---|---|
| BD Pharm Lyse | BD | 555899 |
| Collagenase from Clostridium histolyticum | Sigma-Aldrich | C9407 |
| Hyaluronidase | Sigma-Aldrich | H3506 |

**Histology**

| Reagent | Company | Catalog number |
|---|---|---|
| Cell Conditioning 1 (CC1) | Roche | 950-124 |
| Eosin Y aqueous solution | Sigma | HT110232 |
| Hemalum solution acid acc. to Mayer | Carl Rorth | T865.1 |
| Human Cytokeratin 5/6 (D5/16B4) | Roche | 790-4554 |
| ProLong™ Gold Antifade Mountant with DAPI | Invitrogen | P36931 |
| TissueTek O.C.T. Compound | Sakura | TTEK |
| ultraView Universal DAB Detection kit | Roche | 760-500 |

**Antibodies**

| Reagent | Company | Catalog number |
|---|---|---|
| CLA FITC (HECA-452) | Biolegend | 321306 |
| CLA bv605 (HECA-452) | BD | 563960 |
| CD1a FITC (HI149) | BioLegend | 300104 |
| CD3 PerCP-Cy5.5 (OKT3) | eBioscience | 45-0037-42 |
| CD3 BV421 (UCHT1) | BioLegend | 300434 |
| CD3 bv605 (SK7) | BioLegend | 344835 |
| CD3 PE (HIT3a) | BD | 561803 |
| CD3 PE-Cy5.5 (UCHT1) | eBioscience | 15-0038-42 |
| CD4 PE-594 (RPA-T4) | BioLegend | 300548 |
| CD4 PE-Cy5 (RPA-T4) | BioLegend | 300510 |
| CD4 Alexa Fluor 700 (RPA-T4) | Biolegend | 300526 |
| CD4 bv500 (RPA-T4) | BD | 560768 |
| CD8 PE (OKT8) | eBioscience | 12-0086-42 |
| CD8 Pacific Orange (3B5) | eBioscience | MHCD0830 |
| CD8 bv510 (RPA-T8) | BioLegend | 301048 |
| CD14 bv421 (M5E2) | BioLegend | 301830 |
| CD25 PE-Cy5 | BioLegend | 302608 |
| CD25 PE-Cy7 (BC96) | eBioscience | 302612 |
| CD45 PerCP-Cy5.5 (HI30) | BD | 564105 |
| CD45 APC (HI30) | eBioscience | 17-0459-42 |
| CD45 BV785 (HI30) | BioLegend | 304048 |
| CD45RA PE-e610 (HI100) | eBioscience | 61-0458-42 |
| CD45RAPerCP-Cy5.5 (HI100) | BioLegend | 304122 |
| CD45RA Alexa Fluor 700 (HI100) | BioLegend | 304120 |
| CD62L AF700 (DREG-56) | Biolegend | 304820 |
| CD69 PE (FN50) | BioLegend | 310906 |
| CD69 PerCP/eFluor710 (FN50) | eBioscience | 46-0699-42 |
| CD86 PE (B7-2) | eBioscience | 12-0869-41 |

| | | |
|---|---|---|
| CCR6 PE-Cy7 (G034E3) | BioLegend | 353418 |
| CCR7 bv605 (G043H7) | BioLegend | 353224 |
| GM-CSF bv421 (BVD2-21C11) | BD | 562930 |
| HLA-DR AF700 (L243) | BioLegend | 307626 |
| HLA-DR eFluor 780 (LN3) | eBioscience | 47-9956-42 |
| IFNg PE-Cy7 (B27) | BioLegend | 506518 |
| IFN-g BV605 (4S.B3) | BD | 563731 |
| IFN-g AF700 (4S.B3) | BioLegend | 502520 |
| IL-2 PE (MQ1-17H12) | eBioscience | 12-7029-42 |
| IL17A APC (BL168) | BioLegend | 512334 |
| IL-17A PerCP-Cy5.5 (eBio64DEC17) | eBioscience | 45-7179-42 |
| IL-13 PE (JES10-5A2) | BD | 554571 |
| IL-22 PE-Cy7 (22URTI) | eBioscience | 25-7229-42 |
| IL-22 PE-PE (22URTI) | eBioscience | 12-7229-42 |
| ki67 bv605 (ki67) | BioLegend | 350521 |
| ki-67 PE-Cy7 (SolA15) | eBioscience | 25-5698-82 |
| TNFa FITC (Mab11) | BioLegend | 502915 |
| TNFa PE-594 (Mab11) | BioLegend | 502946 |
| TCRgd PerCP-eFluor710 (B1.1) | eBioscience | 46-9959 |
| Fixable Viability Dye eFluor™ 780 | eBioscience | 65-0865-14 |
| Fixable Viability Dye eFluor™ 520 | eBioscience | 65-0867-14 |
| eBioscience™ Fixable Viability Dye eFluor™ 506 | eBioscience | 65-0866-14 |
| IF primary antibody: rabbit anti-human NC-1 domain of type VII collagen (LH7.2) | kindly provided by Dr. Alexander Nyström, University of Freiburg, Germany | |
| IF secondary antibody: goat anti-rabbit A488 | ThermoFisher | A11008 |
| mLy6G (Gr-1) InVivoMab RB6-8C5 | BioXcell | BE0075 |
| Cell Proliferation Dye eFluor™ 450 | ThermoFisher | 65-0842-85 |
